# Cardiac Manifestations in a Case of Severe Hyperkalemia

**DOI:** 10.7759/cureus.13641

**Published:** 2021-03-01

**Authors:** Syed M Saad, Samiya Yasin, Neeraj Jain, Paul LeLorier

**Affiliations:** 1 Cardiology, Louisiana State University Health Sciences Center, New Orleans, USA; 2 Internal Medicine, JenCare, Metairie, USA; 3 Internal Medicine, Louisiana State University Health Sciences Center, New Orleans, USA

**Keywords:** hyperkalemia, hemodialysis, electrocardiogram

## Abstract

Severe hyperkalemia is a life-threatening electrolyte imbalance that may lead to fatal arrhythmias. ECG (electrocardiogram) and serum potassium levels are vital for diagnosing and stratifying the risk. Management involves shifting potassium intracellularly and eliminating it through renal and gastrointestinal routes. Failure to diagnose early and manage severe hyperkalemia requires emergent hemodialysis.

## Introduction

Hyperkalemia is often referred as “the syphilis of electrocardiography” as it is known to produce variable electrocardiogram (ECG) findings [1]. It is classified into mild hyperkalemia (5.5-6.0 mEq/L), moderate hyperkalemia (6.1-7.0 mEq/L), and severe hyperkalemia (>7.1 mEq/L) [2]. Typically hyperkalemia does not cause symptoms. When severe, it can manifest as palpitations, muscle weakness or paralysis, metabolic acidosis, cardiac conduction abnormalities, and cardiac arrhythmias [1]. The latter include tachyarrhythmias, atrioventricular and bundle branch blocks, conduction delays, sinus arrest, and pseudo-infarct pattern of ST-elevation myocardial infarction [3]. Tall hyperacute T wave, flattened P wave, and prolonged PR and QRS intervals, which in extreme cases assume a sine wave pattern, have been commonly reported [3]. We present a case of severe hyperkalemia with uncommon ECG findings.

## Case presentation

**Figure 1:**
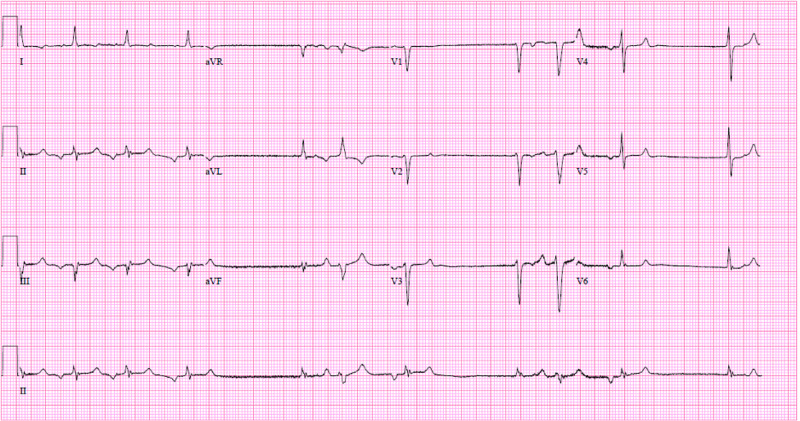
ECG showing sinus arrest with ectopic atrial rhythm and intermittent junctional escape beats.

An 80-year-old female presented with a past medical history of hypertension, type II diabetes mellitus, transient ischemic attack, and end-stage renal disease due to diabetic nephropathy. She had been anuric and on hemodialysis for four years. She presented to the emergency department accompanied by her son due to generalized weakness, dyspnea on exertion, and mild confusion for the past three days. She had missed her last hemodialysis session and denied any chest pain. Her home medicines included aspirin, atorvastatin, amlodipine, carvedilol, hydralazine, and ergocalciferol. Her blood pressure was 131/58 mmHg with an irregularly irregular heart rate of 87 beats per minute. Physical examination demonstrated lethargy, confusion, and volume overload. Laboratory work-up was significant for a potassium level of 8.6 mEq/L and creatinine of 12.2 mg/dL. Repeat potassium was 8.7 mEq/L. Calcium level was normal, and blood gas results were as follows: pH of 7.28, pCO_2_ of 32 mm Hg, pO_2_ of 99 mmHg, and HCO_3_ of 12.8 mmol/L. ECG showed sinus arrest with ectopic atrial rhythm and intermittent junctional escape beats (Figure 1). She was admitted to the intensive care unit for close monitoring of her rhythm and neurologic status.

She had been in sinus rhythm prior to this presentation.

**Figure 2:**
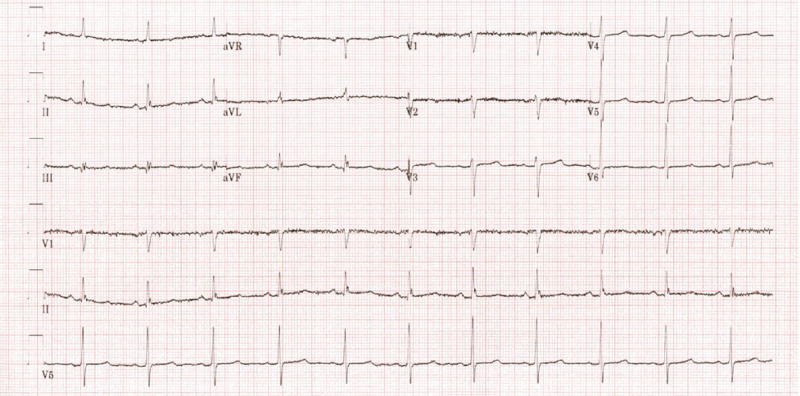
ECG obtained after two sessions of hemodialysis showing normal sinus rhythm with non-specific ST-T wave abnormality.

The patient was given intravenous calcium gluconate, insulin, glucose, and bicarbonate infusion along with potassium-binding resin. Repeat potassium was 8.0 mEq/dL, pH was 7.31, and HCO_3_ was 16.0 mmol/L. There was no improvement in her neurologic status. The nephrology service was consulted and emergent hemodialysis was pursued to manage severe hyperkalemia and metabolic acidosis. Potassium level improved to 5.3 mEq/L, pH to 7.39, and HCO_3_ to 23 mmol/L after the first hemodialysis. Her symptoms improved after two hemodialysis sessions, and hyperkalemia and acidosis resolved. ECG was obtained, which showed normal sinus rhythm with non-specific ST-T wave abnormality (Figure 2).

## Discussion

Pseudohyperkalemia due to hemolysis of blood sample is the most common cause of hyperkalemia, and therefore redrawing a new blood sample is important [4]. Acute or chronic kidney injury, cellular injury from rhabdomyolysis, excessive exercise and other hematologic processes, medications including succinylcholine, non-steroidal anti-inflammatory drugs, potassium supplements, ace inhibitors, and spironolactone, insulin deficiency, diabetic ketoacidosis, and tumor lysis syndrome are other common causes that need to be identified and managed in a timely manner [4].

**Figure 3:**
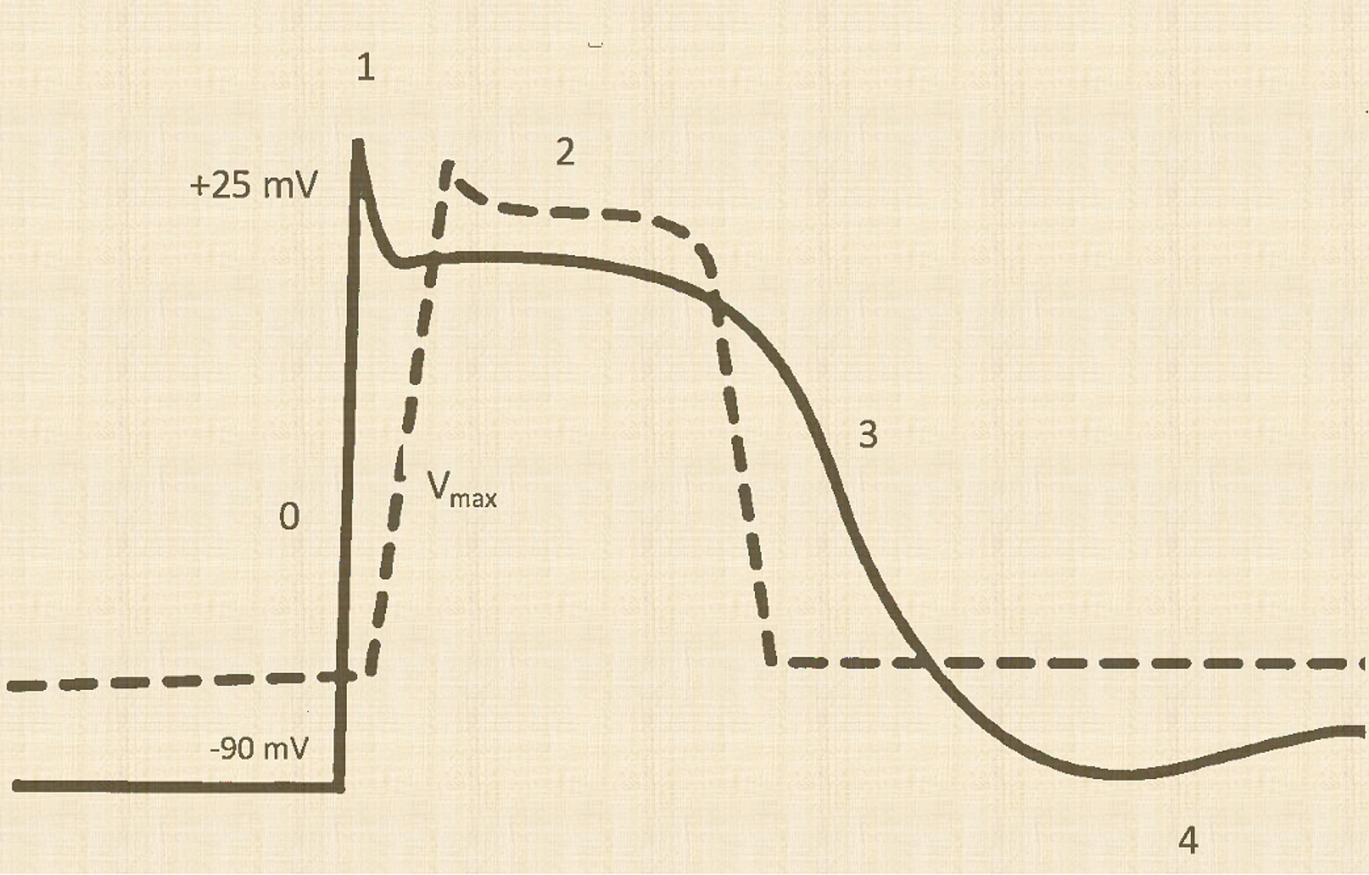
Ventricular action potential in hyperkalemia. In hyperkalemia, the resting membrane potential becomes less negative, and hence fewer sodium channels are available. Consequently, there is a decrease in the rate of rise of phase 0 of action potential, known as Vmax, causing slowing of the rate of impulse conduction, as shown by the dashed line. Also, conductance through IKr increases, resulting in an increase in the potassium efflux from myocytes. There is an increase in the slope of phases 2 and 3 and shortening of repolarization time.

In hyperkalemia, cardiotoxicity can be caused by an increase in resting membrane potential, decreased depolarization, and duration of depolarization [5]. More than 95% of the total body potassium is intracellular. The resting membrane potential becomes less negative in hyperkalemia, and therefore the percentage of available sodium channels decreases. This results in a decrease in the rate of rise of phase 0 of action potential, known as V_max_, causing slowing of the rate of impulse conduction (Figure 3) [6].

IKr potassium channels are responsible for potassium efflux in phases 2 and 3 of the action potential. In hyperkalemia, their conductance increases, resulting in an increase in the potassium efflux from myocytes. Consequently, there is an increase in the slope of phases 2 and 3 and shortening of repolarization time [7] (Figure 3).

Both these mechanisms account for most of the ECG changes mentioned above.

With abnormal depolarization, conduction delays may occur, such as sinoatrial block, and escape rhythms, most commonly junctional [5]. Our patient had findings of sinus arrest with ectopic atrial rhythm and intermittent junctional escape beats at the same time, which is not commonly seen.

It is of paramount importance to identify and manage the ECG changes in hyperkalemia to prevent life-threatening cardiac arrhythmias. Cardiomyocyte stabilization using calcium salt or hypertonic sodium along with insulin administration is first-line therapy in patients with severe hyperkalemia and ECG changes [8]. β2 agonist and sodium bicarbonate in case of metabolic acidosis are second-line therapies [8]. We did not administer β2 agonists as our patient had altered mental status and hypervolemia and was in atrial fibrillation. Strategies increasing potassium renal excretion usually take time and eventually decrease the total potassium pool [9]. If severe hyperkalemia and metabolic acidosis persist despite these interventions, renal replacement therapy or hemodialysis should be undertaken [9].

## Conclusions

Hyperkalemia-related ECG changes should be recognized promptly and warrant emergent medical intervention with cardiac protection and potassium-lowering agents. The patient should be closely monitored for arrhythmias. Emergent dialysis remains definitive treatment for patients with end-stage renal disease and severe hyperkalemia who do not respond to medical management.
